# Early and late effects of the DPP-4 inhibitor vildagliptin in a rat model of post-myocardial infarction heart failure

**DOI:** 10.1186/1475-2840-10-85

**Published:** 2011-09-28

**Authors:** Meimei Yin, Herman HW Silljé, Maxi Meissner, Wiek H van Gilst, Rudolf A de Boer

**Affiliations:** 1 University Medical Center Groningen, University of Groningen, Department of Cardiology, Groningen, The Netherlands

**Keywords:** vildagliptin, myocardial infarction, cardiac remodeling, heart failure, diabetes

## Abstract

**Background:**

Progressive remodeling after myocardial infarction (MI) is a leading cause of morbidity and mortality. Recently, glucagon-like peptide (GLP)-1 was shown to have cardioprotective effects, but treatment with GLP-1 is limited by its short half-life. It is rapidly degraded by the enzyme dipeptidyl peptidase-4 (DPP-4), an enzyme which inhibits GLP-1 activity. We hypothesized that the DPP-4 inhibitor vildagliptin will increase levels of GLP-1 and may exert protective effects on cardiac function after MI.

**Methods:**

Sprague-Dawley rats were either subjected to coronary ligation to induce MI and left ventricular (LV) remodeling, or sham operation. Parts of the rats with an MI were pre-treated for 2 days with the DPP-4 inhibitor vildagliptin (MI-Vildagliptin immediate, MI-VI, 15 mg/kg/day). The remainder of the rats was, three weeks after coronary artery ligation, subjected to treatment with DPP-4 inhibitor vildagliptin (MI-Vildagliptin Late, MI-VL) or control (MI). At 12 weeks, echocardiography and invasive hemodynamics were measured and molecular analysis and immunohistochemistry were performed.

**Results:**

Vildagliptin inhibited the DPP-4 enzymatic activity by almost 70% and increased active GLP-1 levels by about 3-fold in plasma in both treated groups (p < 0.05 vs. non-treated groups). Cardiac function (ejection fraction) was decreased in all 3 MI groups compared with Sham group (p < 0.05); treatment with vildagliptin, either early or late, did not reverse cardiac remodeling. ANP (atrial natriuretic peptide) and BNP (brain natriuretic peptide) mRNA levels were significantly increased in all 3 MI groups, but no significant reductions were observed in both vildagliptin groups. Vildagliptin also did not change cardiomyocyte size or capillary density after MI. No effects were detected on glucose level and body weight in the post-MI remodeling model.

**Conclusion:**

Vildagliptin increases the active GLP-1 level via inhibition of DPP-4, but it has no substantial protective effects on cardiac function in this well established long-term post-MI cardiac remodeling model.

## Introduction

Glucagon-like peptide-1(GLP-1; 7-36 amide), which belongs to the proglucagon family of incretin peptides, is secreted by enteroendocrine L cells of the intestinal mucosa and released in response to food intake [1]. GLP-1 analogues have been used for the clinical treatment of type 2 diabetes because of its multiple actions on pancreatic function [2-4]. Besides its effects on glucose metabolism, GLP-1 has been proven to exert cardiovascular effects in clinical and experimental studies, in the presence or absence of diabetes [5].

GLP-1 receptors (GLP-1R) are expressed in rodent and human heart and vasculature [6-8]. GLP-1R deficient mice exhibit increased left ventricular (LV) thickness, impaired LV contractility and LV diastolic function compared with control mice [9]. However, whether the beneficial effects of GLP-1 on the heart are conferred through direct GLP-1R signaling or indirect, through the GLP-1R-dependent improvement in glucose metabolism is not well established. Administration of GLP-1 improves myocardial function and cardiac output in experimental models of cardiac injury or heart failure. GLP-1 increased cardiac output, and reduced LV end diastolic pressure, in association with improved myocardial insulin sensitivity and myocardial glucose uptake in dogs with rapid pacing-induced congestive heart failure [10]. Consistent with the cytoprotective action of GLP-1 in the endocrine pancreas, GLP-1 reduced infarct size in the isolated perfused rat heart and in animal models of myocardial ischemia [11-13]. A 72 hours infusion of GLP-1 in patients with acute myocardial infarction (MI) and an LV ejection fraction (LVEF) less than 40% resulted in significantly improved LVEF and improved regional and global wall motion scores, in association with a trend towards earlier hospital discharge [14]. In a pilot study of both diabetic and non-diabetic subjects with heart failure, an improved LV function was observed following a 5 week continuous infusion of GLP-1(7-36) [5].

However, active GLP-1 in the circulation is rapidly (within two minutes) degraded by dipeptidyl peptidase-4 (DPP-4) [15]. An alternative approach for enhancing GLP-1 action involves the use of DPP-4 inhibitors. The DPP-4 inhibitor sitagliptin [16] and saxagliptin [17] have been approved for type 2 diabetic patients. Vildagliptin is only approved and used in Europe [18].

The studies on cardiovascular effects of GLP-1, discussed above, have consequently assessed only short-term improvements in cardiac performance, like in post-ischemic or cardiomyopathy states. There are no reports on long-term effects of DPP-4 inhibition in a post-MI cardiac remodeling model. Furthermore, the actions of DPP-4 inhibitors on cardiac remodeling after MI are incompletely understood. We hypothesized that the DPP-4 inhibitor vildagliptin may exert beneficial effects on infarcted hearts by inhibiting the degradation of active GLP-1 and other cardiovascular peptides. The purpose of our study was therefore to determine whether vildagliptin has beneficial effects on long-term post-MI remodeling in rats and to explore the mechanisms underpinning these effects.

## Methods and materials

### Animals

Male Sprague-Dawley rats (Harlan, Zeist, The Netherlands) weighing 250-260 g were housed in groups of 4-5 on a 12-hour light-dark cycle with standard rat chow and water available *ad libitum*. The animals were subjected to sham-surgery or left coronary artery ligation. All experiments were carried out after approval of the Animal Ethical Committee of the University of Groningen for the use of experimental animals and conform to the Guide for Care and Use of Laboratory Animals.

### Drugs

The DPP-4 inhibitor vildagliptin was kindly supplied by Novartis, The Netherlands. Vildagliptin was dissolved in the drinking water and administered in a final concentration of 15 mg/kg/day, which is chosen according to the previous studies [19,20].

### Experimental protocol

Rats were randomly subjected to induction of MI or sham surgery. Briefly, animals were intubated and mechanically ventilated with 2.5% isoflurane in room air enriched with 1.0 L/min oxygen. After left-sided thoracotomy, MI was induced by ligating the proximal portion of the left coronary artery. In sham-operated rats, the same surgery was performed without ligating the suture. Parts of the rats with MI were pre-treated for 2 days with vildagliptin (MI-Vildagliptin immediate, MI-VI). The remainder of the rats was, three weeks after coronary artery ligation, subjected to treatment with DPP-4 inhibitor vildagliptin (MI-Vildagliptin Late, MI-VL) or control (MI). At week 12, cardiac function was determined by echocardiography. After 12 weeks, rats were anaesthetized and hemodynamic function was measured invasively; thereafter blood was drawn (either anticoagulated with EDTA, or left to clot for serum) and the hearts were rapidly excised. Myocardial tissue was sectioned transversally and processed for immunohistochemistry or snap-frozen for molecular analysis.

### Echocardiographic measurements

Cardiac function was assessed by echocardiography by a Vivid 7 (GE Healthcare) equipped with a 10-MHz phase array linear transducer. The echocardiographic measurements were performed under general anaesthesia with 2.5% isoflurane, by a researcher blinded for the treatment allocation. Both 2-dimensional (2D) images in parasternal long-axis and short-axis view and 2-D guided M-mode tracings were obtained. Short-axis views were recorded at the level of mid-papillary muscles. LV internal dimensions in diastole and systole (LVIDd and LVIDs) were measured using M-mode and calculated from three cardiac cycles. LV fractional shortening (FS %) was calculated as follows: FS = (LVIDd-LVIDs)/LVIDd×100%. LV ejection fraction (EF %) was calculated using the Teichholz method.

### Hemodynamic measurements

At sacrifice, rats were anesthetized and a micro-tip pressure transducer (Millar Instr. Inc., Houston, TX, USA) was inserted into the right carotid artery. Arterial systolic and diastolic blood pressures (SBP, DBP) were recorded in the aortic arch. The catheter was advanced into the LV cavity. After a 5-min period of stabilization, heart rate (HR), LV systolic pressure (LVSP), LV end-diastolic pressure (LVEDP), and developed LV pressure (dLVP = LVSP-LVEDP) were measured. For indices of contractility and relaxation, the maximal rates of increase and decrease in LVP *dp/dt*max and *dp/dt*min were determined.

### Procurement of heart tissue and infarct size measurement

After the rats were euthanized, hearts were rapidly excised and arrested in diastole in 2 M ice-cold KCl. The total heart was weighed (heart weight, HW). The right ventricle and atria were removed, and the left ventricle was weighed (left ventricular weight, LVW). The basal and apical parts of the LV were snap-frozen in liquid nitrogen. A mid-papillary slice of the LV was fixed in 4% paraformaldehyde overnight and paraffin-embedded. Paraffin blocks were sectioned, and slides were dehydrated. The 5 μm sections were stained with picrosirius red/fast green [21]. The infarct size was calculated as percentage of the scar length to the total LV circumference. The images were obtained with a Leica microscope and analyzed using appropriate software (Image-pro plus, version 4.5.0.29).

### Biomolecular assays

EDTA plasma was used to measure active GLP-1 and DPP-4 activity, using a commercial Enzyme Linked Immunosorbent Assay, according to the guidelines provided by the manufacturer (Quantikine, R&D system, London, UK). Glucose levels were measured with a blood glucose monitor (Accu-Check^®^, Roche, Germany). Plasma triglyceride and cholesterol levels were determined using commercially available kits (Roche Diagnostics, Mannheim, Germany and DiaSys Diagnostic Systerms, Holzheim, Germany).

### Capillary density

To visualize the capillaries in the myocardium, endothelial cells were stained with Lection GSI (Sigma-Aldrich Chemie, Zwijndrecht, The Netherlands), as previously described [22]. Briefly, 5-μm sections were deparaffinised and rehydrated and endogenous peroxidase was inhibited by methanol/H_2_O_2 _(0.3%) for 15 minutes. Sections were incubated overnight with biotinylated Lectin GSI (1:100) at room temperature. Then, in a second step, the signal was intensified with an ABC-complex containing peroxidase labeled biotins (1:100) (Lab vision, CA, USA). Finally, the sections were incubated with a Ni-Co amplified DAB solution to which a stable peroxide substrate buffer was added (Pierce, CA, USA). Endothelial cells of capillaries and larger vessels were visualized in the myocardium as a brown precipitate. A background staining was not used in order to avoid interference with the Lectin staining. Capillary density in the viable LV wall was calculated as the number of capillaries per tissue area.

### Left ventricular hypertrophy

First, left ventricular hypertrophy was expressed as the ratio of left ventricular weight to body weight. Microscopically, left ventricles were cut into 5 μm transversal slices from apex to base. Afterwards, sections were stained with a Gomori's silver staining in order to visualize the membrane of the cardiomyocytes, as described previously [21]. Cardiomyocyte size was measured transversally cut in the border zone of the infarcted area using image analysis (Zeiss KS400, Germany).

### Real-time PCR

We used myocardial tissue (of border zone of the infarction) to extract total RNA with TRIzol reagent (Invitrogen Corp., Carlsbad, CA, USA). The nano-drop device was used to quantify the RNA concentration. First strand cDNA was synthesized by reverse transcription reaction by using random primer mix, and used as a template to amplify genes of interest; for this specific primers against ANP (atrial natriuretic peptide), BNP (brain natriuretic peptide), collagen I, MMP-9 (matrix metalloproteinase-9) were designed. All gene expression values were normalized to 36B4 mRNA levels.

### Data analysis

All data are presented as means ± standard errors of the mean (sem). Data of infarcted rats were only included if the infarction comprised the major part of the LV free wall, since small infarctions (< 20%) are found to be hemodynamic fully compensated (two animals were excluded from the MI group, and three animals were excluded from each vildagliptin-treated groups MI-VI and MI-VL). Statistical analysis among groups was performed by one-way analysis of variance (ANOVA) followed by Tukey post-hoc test. Within-group comparisons between week 3 and week 12 were analyzed by Paired-Sample T-Test. Differences were considered statistically significant if p < 0.05.

## Results

### Active GLP-1, DPP-4 activity and glucose levels

**Figure 1:**
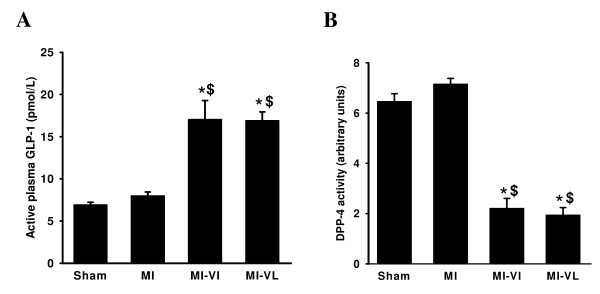
**Effects of Vildagliptin on DPP-4 activity and active GLP-1 levels**. A:Vildagliptin increases active plasma GLP-1 levels after 12 weeks treatment. B: Vildagliptin reduces DPP-4 activity after 12 weeks treatment.Data are presented as means **± **SEM. N = 8-10 for all groups. *P < 0.05 vs. sham group; $P < 0.05 vs. MI group.

We measured the DPP-4 activity and active GLP-1 levels in plasma after 12 weeks of vildagliptin treatment to confirm that continuous administration of vildagliptin (supplied at 15 mg/kg in the drinking water) was indeed associated with suppression of plasma DPP-4 activity, and increase in the active GLP-1 levels. Plasma DPP-4 activity was significantly reduced by almost 70% in both treatment groups compared to non-treated groups. This was associated with a significant increase of active GLP-1 level in both vildagliptin groups (Figure 1).

During the 12 weeks of follow up, we measured glucose levels at baseline, week 6 and week 12. No significant changes in glucose levels were observed, suggesting that vildagliptin has no effect on normal glucose levels. The similar findings were observed in plasma cholesterol and triglycerides that no significant differences were found among all groups (Table 1B).

**Table 1:** Characteristics of the experimental groups at sacrifice (12 weeks).

		Sham N = 8	MI N = 10	MI-VI N = 9	MI-VL N = 9
A	BW (g) (baseline)	321 ± 9	313 ± 5	321 ± 2	306 ± 4
	BW (g) (12 week)	446 ± 15	449 ± 11	436 ± 5	450 ± 7
	LVW (g) (12 week)	0.93 ± 0.03	1.05 ± 0.03*	0.97 ± 0.02	1.08 ± 0.03*
	LVW/BW	2.00 ± 0.05	2.34 ± 0.09**	2.22 ± 0.05	2.39 ± 0.05**

B	Glucose (mmol/L) (3 week)	9.0 ± 0.6	8.0 ± 0.5	7.8 ± 0.3	9.5 ± 0.4
	Glucose (mmol/L) (6 week)	7.2 ± 0.2	7.3 ± 0.3	7.1 ± 0.1	7.7 ± 0.2
	Glucose (mmol/L) (12 week)	8.0 ± 0.3	7.3 ± 0.2	8.0 ± 0.3	7.6 ± 0.2
	Cholesterol (mmol/L)	1.49 ± 0.09	1.48 ± 0.04	1.45 ± 0.08	1.39 ± 0.07
	Triglycerides (mmol/L)	0.95 ± 0.19	1.12 ± 0.13	0.84 ± 0.11	0.86 ± 0.12

Data are presented as means **± **SEM; N indicates number of animals; BW, body weight; LVW, left ventricle weight; LVW/BW, left ventricle weight/body weight ratio.**P < 0.01 vs. Sham.*P < 0.05 vs. Sham.

### Body Weight, LV weight, and infarct Size

**Figure 2:**
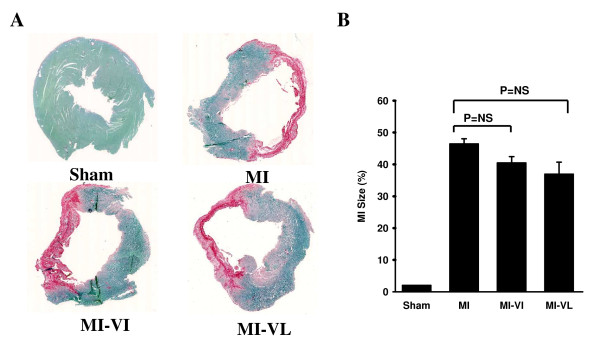
**Effects of Vildagliptin on infarct size. **A: Sirius red and fast green staining of rat myocardium shows the infarcted area with the red color in all groups. B:Graphic representation of infarct size expressed as percentage of the scar length to the total LV circumference. Data are presented as means ±SEM. N = 8-10 for all groups.

Body weight (BW), LV weight (LVW) to body weight ratios (LVW/BW) and infarct sizes are shown in Table 1A. Ligation of the left coronary artery resulted in an infarct size of 47 ± 2% in control-treated MI group (Figure 2B). Although there was no significant reduction in the infarct size, vildagliptin treatment (both immediate and late) was associated with a trend towards smaller infarct size over the 3-month period of treatment compared with the MI control group (Figure 2B). There was no difference in body weight between groups both at baseline and at 12 weeks after MI surgery. The LVW/BW ratios in all MI groups were significantly increased compared to LVW/BW of the sham-operated group, however, LV hypertrophy was not significantly reduced by vildagliptin treatment (both groups) compared to MI control (Table 1A).

### LV hemodynamic and echocardiographic parameters

All MI rats showed evidence of heart failure, including development of LV dilatation and LV systolic dysfunction at both 3 weeks and 12 weeks after MI. Three weeks after MI, there was a significant decrease in all MI groups in FS and LVEF compared with the sham-operated group, as well as an enlarged chamber size (LVID) and a thinner wall thickness (IVS) in both systole and diastole (Table 2). During the following 9 weeks, a continued LV chamber enlargement and interventricular septum wall thinning was observed in all MI groups compared with the sham group. Although most echocardiographic parameters were not significantly different between the MI control group and the vildagliptin-treated groups, comparisons between 3 weeks and 12 weeks echocardiographic studies demonstrated that the progressive LV dilatation and wall thinning in the MI control group was attenuated in the vildagliptin-treated groups (Table 2). The LVEF in MI control group decreased from 49 ± 3% at 3 weeks to 36 ± 5% at 12 weeks, while in the early vildagliptin it decreased from 48 ± 4% to 45 ± 5%, and in the late vildagliptin group from 49 ± 3% to 48 ± 4% (Table 2). The differences in LVEF at 12 weeks between the MI-control and the MI-vildagliptin groups were not significant. Thus, a significant worsening of LVEF was observed in the MI control group as compared to both vildagliptin groups.

**Table 2:** Echocardiographic parameters of the experimental groups at week 3 and week 12.

	Week 3				Week 12			
	**Sham**	**MI**	**MI+VI**	**MI+VL**	**Sham**	**MI**	**MI+VI**	**MI+VL**
		
	**N = 8**	**N = 10**	**N = 9**	**N = 9**	**N = 8**	**N = 10**	**N = 9**	**N = 9**

HR (bpm)	377 ± 11	379 ± 9	383 ± 11	407 ± 11	370 ± 12	387 ± 6	377 ± 10	391 ± 6
IVSd(mm)	1.5 ± 0.1	0.9 ± 0.1**	1.0 ± 0.01*	1.0 ± 0.1*	1.6 ± 0.1	0.8 ± 0.1**	1.0 ± 0.1*	0.8 ± 0.1**
LVIDd(mm)	7.7 ± 0.1	8.3 ± 0.3	8.4 ± 0.2	8.1 ± 0.3	8.0 ± 0.4	10.1 ± 0.3** §	9.7 ± 0.4** §	9.7 ± 0.2 ** §
LVPWd(mm)	1.8 ± 0.1	1.8 ± 0.1	1.9 ± 0.1	1.8 ± 0.1	2.1 ± 0.2	2.3 ± 0.2	2.0 ± 0.12	2.0 ± 0.1
IVSs(mm)	2.9 ± 0.1	1.3 ± 0.2*	2.0 ± 0.6	1.3 ± 0.2*	2.9 ± 0.2	1.0 ± 0.2**	1.2 ± 0.2**	1.2 ± 0.2**
LVIDs(mm)	4.2 ± 0.1	6.5 ± 0.3**	6.0 ± 0.4**	6.3 ± 0.3**	4.6 ± 0.4	8.6 ± 0.5** §	7.7 ± 0.4**	7.6 ± 0.2** §
LVPWs(mm)	2.9 ± 0.1	2.7 ± 0.1	2.6 ± 0.2	2.5 ± 0.1	2.9 ± 0.2	2.7 ± 0.3	2.6 ± 0.1	2.6 ± 0.2
LVEF (%)	82 ± 1	49 ± 3**	48 ± 4**	49 ± 3**	78 ± 3	36 ± 5** §	45 ± 5**	48 ± 4**
FS (%)	46 ± 2	22 ± 2**	21 ± 2**	22 ± 2**	43 ± 3	16 ± 3**§	21 ± 2**	22 ± 2**

Data are presented as means ** ± **SEM; N indicates number of animals; bpm, beats per minute. **P < 0.01 and *P < 0.05 indicate significant differences compared to sham-operated rats in week 3 and week 12 respectively; § P < 0.05 indicates significant difference between parameters at week 12 and week 3 using Paired-Sample T-Test. IVSd and IVSs, the thickness of the interventricular septum in diastole and systole; LVIDd and LVIDs, diastolic and systolic left ventricle dimensions; LVPWd and LVPWs, the thickness of left ventricle posterior wall in diastole and systole.

Hemodynamic abnormalities were characteristic for rats with HF in the MI control group. Specifically, MI control rats showed significantly depressed dp/dtmax and elevated LVEDP compared with sham operated rats, while early and late vildagliptin treatment did not result in significant improvement (Table 3).

**Table 3:** Hemodynamic parameters of the experimental groups at sacrifice (12 weeks).

	Sham N = 8	MI N = 10	MI-VI N = 9	MI-VL N = 9
Heart rate (bpm)	346 ± 16	378 ± 7	350 ± 9	361 ± 12
LVESP (mmHg)	117 ± 4	109 ± 3	108 ± 3	109 ± 4
LVEDP (mmHg)	6.2 ± 0.8	9.6 ± 1.6*	9.8 ± 1.3*	7.4 ± 0.5
SBP (mmHg)	115 ± 3	111 ± 2	110 ± 3	113 ± 3
DBP (mmHg)	87 ± 2	86 ± 2	85 ± 2	84 ± 2
dPdtmax (mmHg/s)	8744 ± 435	7245 ± 436	6908 ± 396	7952 ± 484
dPdtmin (mmHg/s)	-9363 ± 268	-6419 ± 466*	-6592 ± 613*	-7077 ± 380*

Data are presented as means ** ± **SEM; N indicates number of animals; LVESP, left ventricular end-systolic pressure; LVEDP, left ventricular end-diastolic pressure; SBP, systolic blood pressure; DBP, diastolic blood pressure; *dP/dt*max and *dP/dt*min, the maximal rate of increase and decrease of left ventricular pressure, respectively.*P < 0.05 vs. Sham.

### Capillary density and cardiomyocyte size

**Figure 3:**
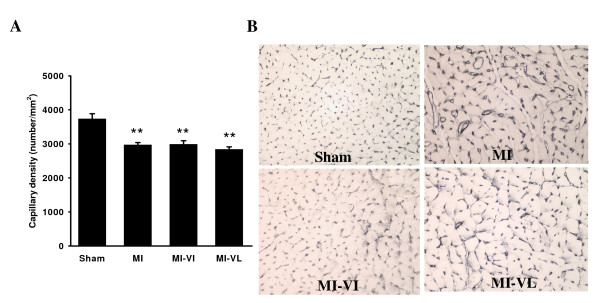
**Effects of vildagliptin on capillary density in cardiomyocyte cross-sectional area**. A: Graphic representation of capillary density expresses as the number of capillaries/mm^2 ^field. B: Typical examples of capillary density in the experimental groups at ×40 magnification. Data are presented as means ** ± **SEM. N = 8-10 for all groups. **P < 0.01 vs. sham group.

**Figure 4:**
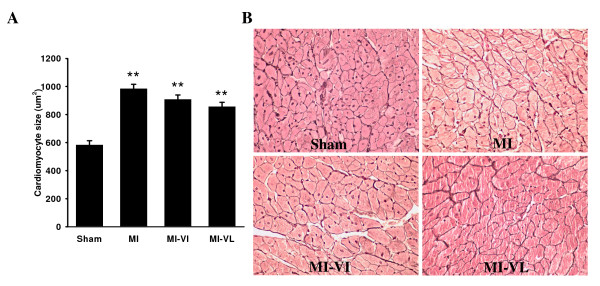
**Cardiomyocyte size was measured by gomori staining in cross-sectional areas**. A: Graphic representation of cardiomyocyte size in all experimental groups. B: Typical examples of cardiomyocyte size in the experimental groups at ×40 magnification. Data are presented as means ** ± **SEM. N = 8-10 for all groups. **P < 0.01 vs. sham group.

Capillary density was significantly reduced in all post-MI groups compared to sham-operated rats (p < 0.01) (Figure 3). Cardiomyocyte size significantly increased in all post-MI groups compared to sham (p < 0.01). Vildagliptin treatment both in early and late phase led to a trend towards diminished cardiomyocyte hypertrophy but no statistically significant effects were found compared to the MI control group ((Figure 4, p = NS).

### Cardiac gene expression

**Figure 5:**
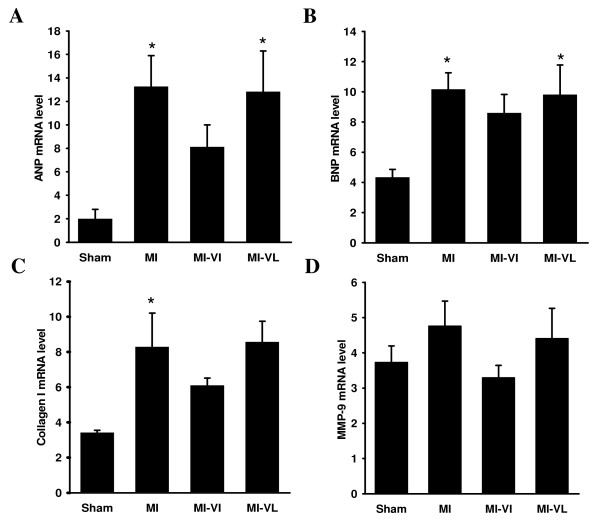
**Quantitative real-time reverse-transcriptase polymerase chain reaction (RT-PCR) was conducted and expression was measured of myocardial atrial natriuretic peptide (ANP; A), brain natriuretic peptide (BNP; B), collagen I (C), and matrix metalloproteinase-9 (MMP-9; D), respectively (mRNA corrected for 36B4 mRNA level)**. The relative corrected values are shown for each group. Data are presented as means ** ± **SEM. N = 8-10 for all groups.*P < 0.05 vs. sham group.

ANP and BNP mRNA levels were significantly increased both in the MI control group as well in as late vildagliptin treatment group compared to sham group (p < 0.05). Vildagliptin treatment both in early and late phase had no significant effects herein (Figure 5A and 5B). Similar results were found for collagen I expression, which was significantly increased in the MI group compared to sham, while no difference was observed in collagen I expression between vildagliptin-treated groups and the control MI group (Figure 5C). MMP-9 mRNA levels were similar in all groups (Figure 5D).

## Discussion

In the present study, we demonstrate that long-term treatment of the DPP-4 inhibitor vildagliptin in rats with LV remodeling due to MI increases endogenous active plasma GLP-1 levels, via inhibition of DPP-4 activity. However, this did not result in a decreased infarct size nor did it attenuate cardiac remodeling associated with post-MI. No differences in glucose levels and body weight were found in these non-diabetic rats when treated with vildagliptin.

To our knowledge, this is the first study to assess the effects of immediate and late vildagliptin treatment in a non-diabetic rat model. We furthermore aimed to dissect immediate versus late effects of DPP-4 inhibition. We herein show that neither early nor late vildagliptin treatments exert beneficial effects in MI-induced deterioration in cardiac remodeling. Notably, MI size was (non-significantly) smaller in the vildagliptin treated rats. However, this effect was observed both in rats immediately treated with vildagliptin as well as in rats in which treatment with vildagliptin started after 3 weeks, so when the infarction scar is fully organized. From this, we postulate that vildagliptin is unlikely to reduce infarct size. The associated differences in LVEF may be ascribed to the differences in infarct size. Other measures of LV remodeling were also unaffected by vildagliptin. For example, both early and late vildagliptin treatment did not reverse the MI-induced decrease in capillary density, measured at the border zone of the infarcted myocardium. Similarly, both early and late vildagliptin treatment did not counteract MI-induced cardiomyocyte hypertrophy. Furthermore, both early and late vildagliptin treatment had no effects on the MI-induced increases in cardiac expression of ANP, BNP. ANP and BNP cardiac gene expression closely associates with the severity of LV dysfunction [23], so this finding supports the notion that cardiac remodeling is not affected by vildagliptin. Furthermore, the expression of matricellular proteins collagen I and MMP-9 were also not affected by vildagliptin treatment.

A number of acute studies have been previously performed with DPP-4 inhibitors, GLP-1 or GLP-1 analogues, addressing their role in cardioprotection. A study with the DPP-4 inhibitor PFK275-055, a vildagliptin-analogue, showed a reduced infarct size with activation of the cardioprotective RISK (reperfusion-induced salvage kinase) pathway in pre-diabetic rats [24], whereas a study with the DPP-4 inhibitor sitagliptin showed that infarct size or short-term cardiac function were not affected by the treatment [25]. The latter study is in line with our study.

Another mean of increasing GLP-1 is direct GLP-1 infusion. Acute GLP-1 infusion studies both in patients and rodents did show beneficial effects. In a clinical study, 3-day infusion of GLP-1 improved LV function in patients after acute MI [14]. Moreover, ischemia/reperfusion experiments in rats showed that GLP-1 administration prior to the ischemia leads to smaller infarct size in the isolated heart [11,12,26]. Another ischemia-reperfusion study showed that only the GLP-1 analogue exendin-4, but not GLP-1(9-36) amide exerts infarct-limiting action, while both of them improved LV performance [27]. Apparently, GLP-1 infusion provides a stronger effect than DPP-4 inhibition, probably due to a stronger elevation of GLP-1 levels. The published results from DPP-4 inhibition studies are variable and this might be explained by different levels of inhibition (different inhibitors used, different dosages), and variable levels of GLP-1.

So, although a number of *acute *studies have been performed, only few *chronic *studies have addressed the effects of GLP-1 on cardiac function in non-diabetic models. A chronic (3 month) infusion study showed that GLP-1 improved LV systolic function and prolonged survival in spontaneously hypertensive rats by increasing myocardial glucose uptake and reducing myocyte apoptosis [28]. Treatment with either GLP-1 or exenatide analogue AC3174 also demonstrated promising cardioprotective effects, including improved LVEF, LV end-diastolic pressure, and cardiac dimensions in a rat MI model (comparable to the present model) [29]. Our results are not in concert with the observations that cardioprotection is achieved by GLP-1 treatment. The reasons for the discrepancies between the present study and earlier work may be attributable to the dosing regimen, the different analogue utilized, the levels of GLP-1 achieved, and the timing of the treatment and the species studied [30].

Furthermore, a number of other issues should be considered when discussing the lack of vildagliptin-induced cardioprotection as observed in our study. First, we used a model of non-diabetic rats, with normal glucose levels. Vildagliptin is an antidiabetic agent which exerts its beneficial effects in the cardiovascular system through glycometabolic control. Thus it may be possible that vildagliptin is more beneficial in diabetic models. Second, as a DPP-4 inhibitor, vildagliptin inhibits degradation of GLP-1 and prolongs its half life *in vivo*; however, its effects are less strong than continuous GLP-1 infusion. The elevations in GLP-1 levels achieved by vildagliptin are likely to be lower than achieved by GLP-1 infusion and thus insufficient to produce cardioprotection. Finally, we do not know if other, hitherto unknown mechanisms, may underpin the effects of vildagliptin in the heart. To date, limited data are available describing the effects of vildagliptin on the cardiovascular system.

In addition, the biological effects of DPP-4 inhibitors appear different from GLP-1 and other GLP-1R agonists [31]. Vildagliptin treatment has no effect on body weight, food intake, energy expenditure and insulin sensitivity [4,32,33]. Although it has been shown that vildagliptin reduced plasma cholesterol and triglycerides in diabetic patients [34,35], no effects of vildagliptin on these parameters were found in our post-MI animal study. However, GLP-1 inhibits glucagon secretion and controls body weight by decreasing food intake, increase insulin sensitivity and improve glucose uptake, which are beneficial cardiovascular factors [36-38]. Moreover, the protective effects of GLP-1 are also mediated through GLP-1R-independent pathways, partially though beneficial effects of its metabolite GLP-1 (9-36) [39]. These differences might explain why we observed only limited improvements in our study with vildagliptin as compared to other studies with GLP-1 and GLP-1 analogues.

## Limitations

Possibly, the dosage of vildagliptin was not sufficient to observe a cardioprotective effect of vildagliptin. We did not include a group treated with GLP-1 infusion so we cannot compare DPP-4 inhibition to GLP-1. Furthermore, although plasma glucose was comparable in all groups, we did not assess factors associated with myocardial glucose metabolism, so that we cannot rule out if direct metabolic effects explain the results.

## Conclusions

Long-term treatment with the DPP-4 inhibitor vildagliptin, started immediate or late after MI, does not preserve cardiac function in a rat post-MI remodeling model of chronic heart failure despite increases in plasma active GLP-1 levels by inhibiting DPP-4 activity.

## Competing interests

The authors declare that they have no competing interests.

## Authors' contributions

MY carried out the animal experiments, the biomolecular studies and drafted the manuscript. HHWS performed molecular studies, and provided important intellectual input to the manuscript. MM performed statistical analysis and provided important intellectual input to the manuscript. WHvG conceived of the study and provided important intellectual input to the manuscript. RAdB conceived of the study, and participated in its design and coordination, and provided important intellectual input to the manuscript. All authors read and approved the final manuscript.
